# Comparative dataset on the characterization of natural polymers and nanocomposites for enhanced oil recovery

**DOI:** 10.1016/j.dib.2020.106506

**Published:** 2020-11-06

**Authors:** Akinleye Sowunmi, Oyinkepreye Orodu, Vincent Efeovbokhan, Solomon Ogundare

**Affiliations:** a Department of Chemical Engineering, Covenant University, P.M.B 1023, Ota, Ogun State, Nigeria; b Department of Petroleum Engineering, Covenant University, P.M.B 1023, Ota, Ogun State, Nigeria

**Keywords:** Polymer, Viscosity, Nanoparticles, Nanocomposites, Enhanced oil recovery, Rheology

## Abstract

Polymer flooding is one of the most effective processes to improve crude oil recovery. However, the capacity of natural polymers to displace crude oil is determined by their rheological behaviour in the face of prevailing reservoir conditions. Poor rheological stability of water-soluble polymers challenges their application in harsh reservoir conditions, making it important to investigate the characteristics of polymers and their corresponding nanocomposites for use in enhanced oil recovery (EOR). The main objective of this work is to conduct characterization tests for three polymers (Gum Arabic, Xanthan Gum and Guar Gum) and three nanoparticles (silica, alumina and cupric), and to investigate the viscosity profile of the polymers under different conditions of temperature, salinity, nanoparticle weight percentage and polymer weight percentage. SEM was used to characterize the nanoparticles while FTIR and TGA were used to characterize the polymers. All viscosity measurements were conducted using an OFITE Viscometer. The SEM, FTIR and TGA results are presented in figures while the viscosity results are presented as raw data in tables. The data should be used to support oil recovery experiments, economic analysis of the use of polymers and nanocomposites in EOR and the study of adsorption and permeability impairment in core flooding tests.

## Specifications Table

**Table:** 

Subject	Petroleum Engineering
Specific subject area	Characterization and measurement of viscosity
Type of data	Tables and Figures
How data were acquired	Brucker Vertex 80v FTIR Instrument, TA Q6000 Instrument for TGA, Quanta SEM 450 Equipment, and Model 800 OFITE Viscometer
Data format	Raw
Parameters for data collection	The following were considered: types of natural polymers, the concentration of natural polymers, types and concentration of nanoparticles, salinity and temperature effect on the viscosity of polymers
Description of data collection	Characterization of the polymers and nanoparticles was done using TGA, FTIR and SEM. Functional groups of the polymers were obtained using FTIR. The thermal stability of the polymers was examined using TGA, and micrographs of the nanoparticles were obtained using SEM. The viscosity of the polymers was measured using a Model 800 OFITE Viscometer and a heating mantle. The effects of polymer concentration, nanoparticle concentration, nanoparticle types, temperature and salinity on the viscosity of three natural polymers were investigated.
Data source location	Department of Petroleum Engineering, Covenant University, Ota, Nigeria
Data accessibility	Data is with the article.

## Value of the Data

• Characterization and rheological study of polymers and nanocomposites make it possible to determine the suitability of these polymers for core flooding experiments and to make a comparison between them based on the conditions typically found in a reservoir formation.
• This data provides significant insight for researchers and industry experts working in the area of chemical enhanced oil recovery processes.
• This data provides a basis for the use of polymers and nanocomposites for core flooding experiments with a view to reducing the possibility of permeability impairment from the viscosity of the polymers and agglomeration of the nanocomposites. The combined use of polymers and nanoparticles make it possible to achieve higher oil recoveries from an increase in volumetric and microscopic efficiencies.

## 1 Data Description

**Fig. 1:**
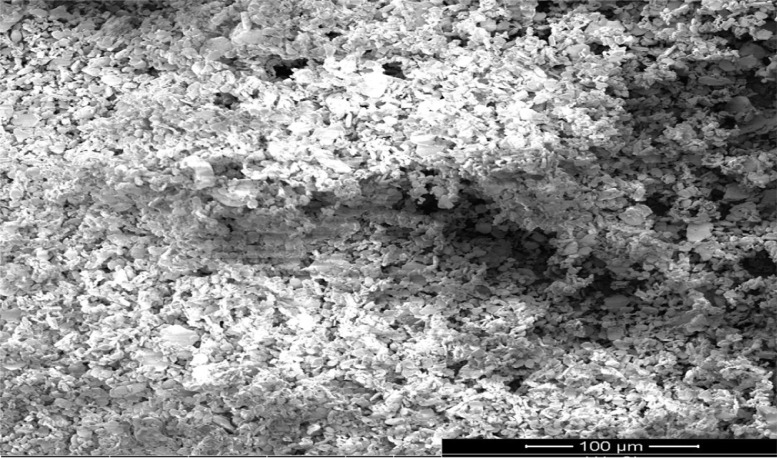
SEM image of silicon oxide nanoparticles.

**Fig. 2:**
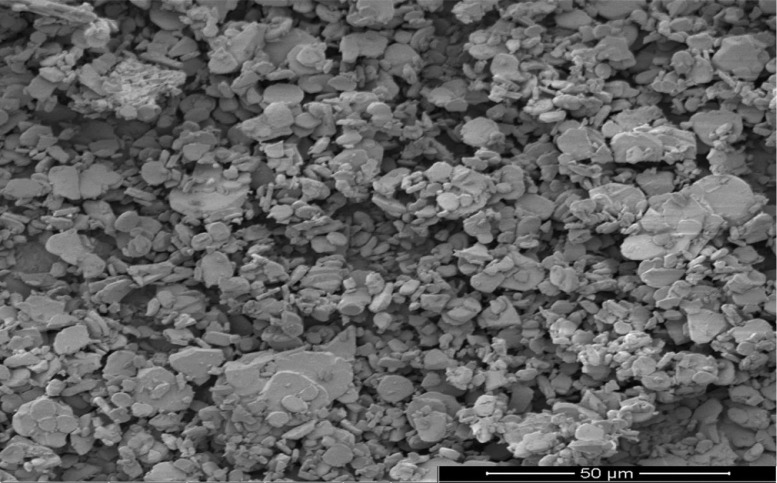
SEM image of aluminium oxide nanoparticles.

**Fig. 3:**
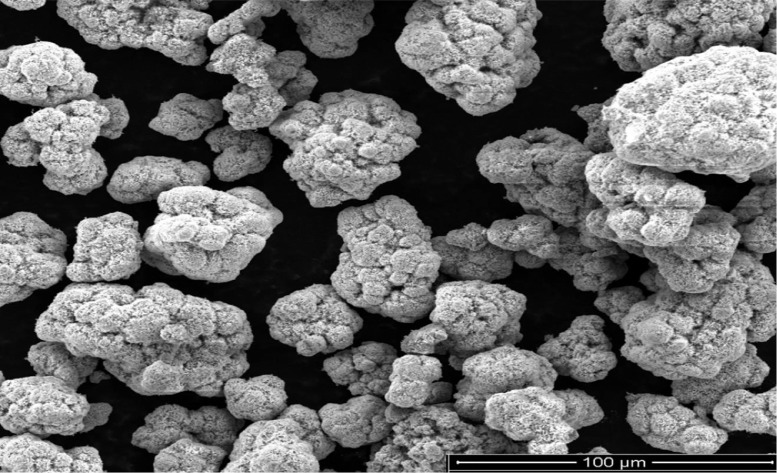
SEM image of cupric oxide nanoparticles.

**Fig. 4:**
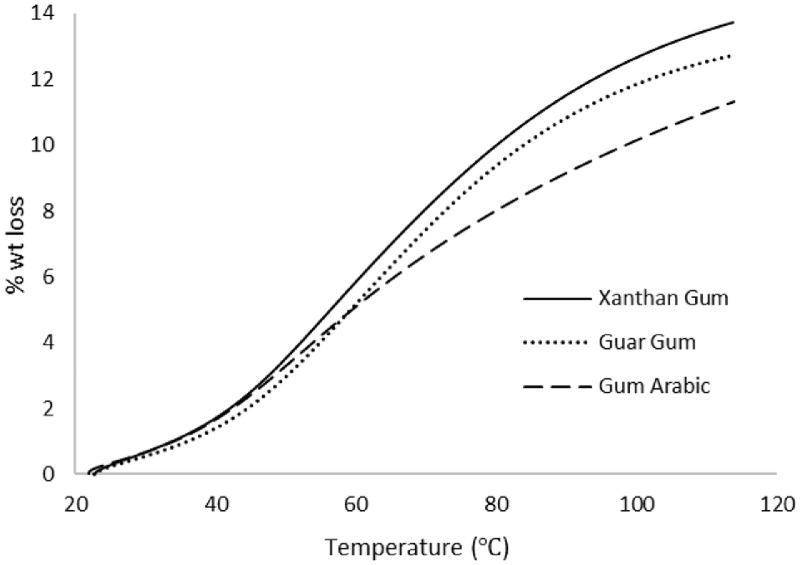
Thermogravimetric analysis for polymers.

**Fig. 5:**
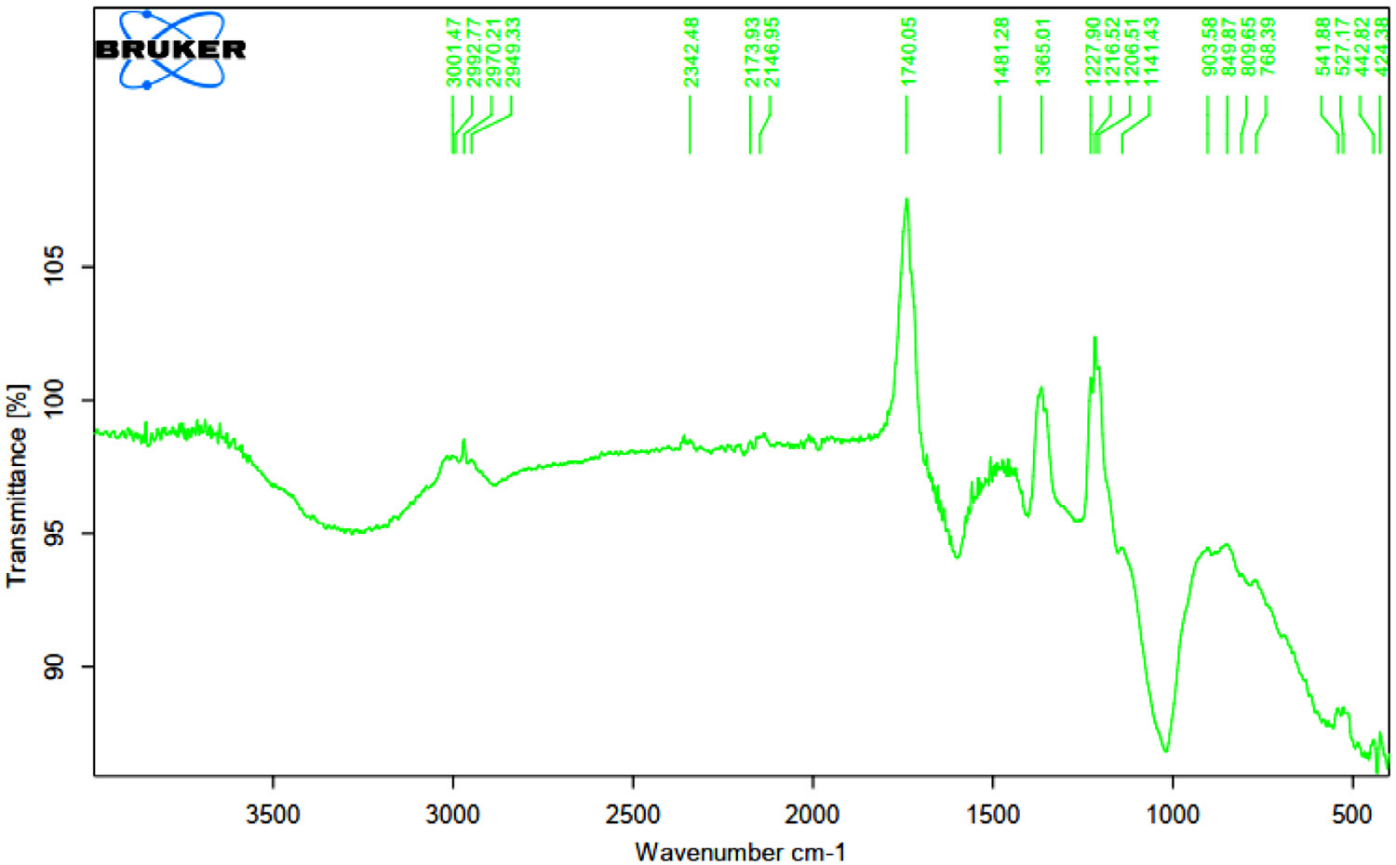
FTIR spectra for xanthan gum polymer.

**Fig. 6:**
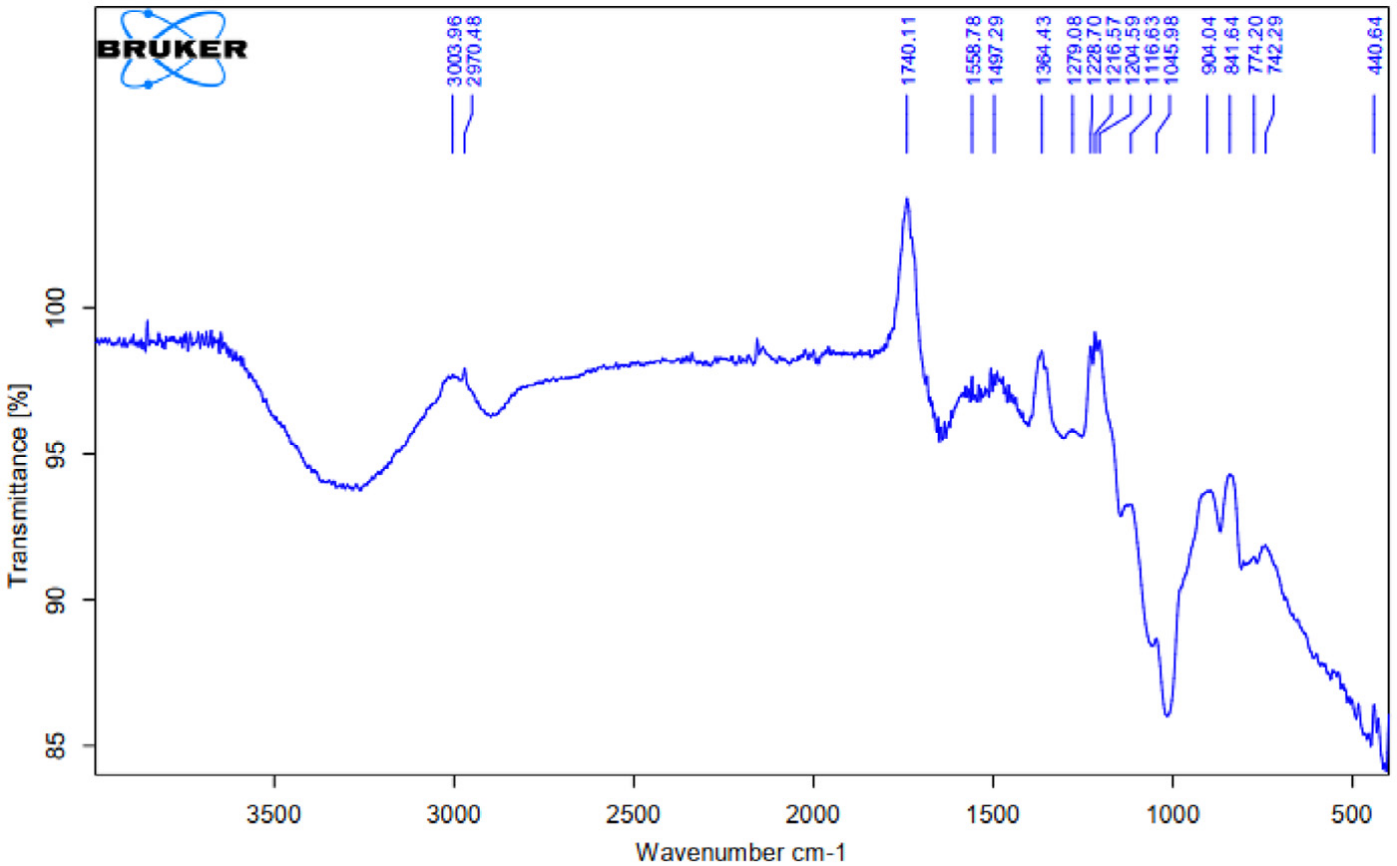
FTIR spectra for guar gum polymer.

**Fig. 7:**
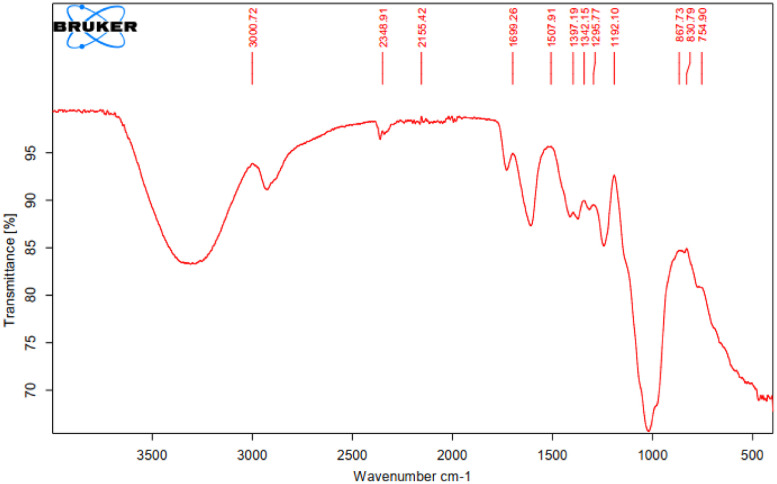
FTIR spectra for gum arabic polymer.

As oil reservoirs around the world decline in crude oil production, several options exist for recovering more crude oil from reservoirs with waning production [1]. Polymers have been reported to enhance oil recovery by improving the volumetric efficiency of the flooding process [2], [3], [4], [5]. However, polymers alone are incapable of microscopic interactions, thereby limiting their oil recovery capacity. It is therefore important to investigate the stability of polymers and nanocomposite mixtures under existing reservoir conditions for potential use in EOR. The dataset presented in this article shows the characterization of polymers and nanoparticles using a scanning electron microscope (SEM), Fourier-transform infrared spectroscopy (FTIR) and thermogravimetric analysis (TGA). Also, the effects of polymer weight percentage, nanoparticle weight percentage, nanoparticle type, temperature and salinity on the viscosity of polymers were investigated. Three nanoparticles were studied: silica, alumina and cupric nanoparticles. The SEM micrographs of these nanoparticles are shown in Figs. 1–3. Three natural polymers were also studied: xanthan gum, guar gum and gum arabic. The thermal stability of the polymers was measured using TGA; the plots of the percentage weight losses against temperature are shown in Fig. 4, and the raw data obtained from the TGA machine are provided in Table 15, Table 16, Table 17. The FTIR spectra for xanthan gum, guar gum and gum arabic are shown in Figs. 5–7; while the list of functional groups is shown in Table 1. The effect of temperature and polymer weight percentage at different shear rates on the viscosities of xanthan gum and guar gum are shown in Tables 2 and 3 respectively. The temperatures used for the viscosity experiments were 30, 50, 75, and 90 °C; while the weight percentages used for the polymers were 0.1, 0.2, 0.3, 0.4, 0.5, and 1% w/w. Table 4 shows the effect of temperature and weight percentage on the viscosity of gum arabic. The same temperatures were used for gum arabic viscosity measurements, but the weight percentages used were 0.4, 0.5, 1, 5, 10 and 15% w/w. Higher weight percentages were used for gum arabic due to the practical limitations of using lower weight percentages owing to the low viscosity of gum arabic polymer. The effect of silica, alumina and cupric loading on xanthan gum viscosity at different shear rates are shown in Table 5, Table 6, Table 7. The effects of these nanoparticles on guar gum and gum arabic polymers are shown in Table 8, Table 9, Table 10, Table 11, Table 12, Table 13 respectively. The effect of salinity on the viscosities of xanthan gum, guar gum and gum arabic is shown in Table 14.

**Table 1:** Functional groups contained in polymers.

Polymers	Wavelength (cm^−1^)	Functional groups
Xanthan gum	3263	O–H stretching
	2883	C–H stretching
	1739	C=O stretching
	1598, 1404	COO^–^ symmetric stretching
		COO^–^ asymmetric stretching
		O–H angular deformation
	1250	C=O deformation
	1018	C–O stretching
Guar gum	3261	O–H stretching vibration
	2885	C–H stretching
	1648	Ring stretching
	1402	CH_2_ symmetric deformation
	1143	C–OH stretching
	1053	CH_2_OH stretching
	1014	CH_2_ twisting vibration
	750-500	Carbohydrate fingerprint
Gum arabic	3261	O–H stretching
	2885	C–H stretching
	1641	COO^–^ symmetric stretching
	1402	COO^–^ asymmetric stretching
	1250-900	Carbohydrate fingerprint

**Table 2:** Xanthan gum viscosity (cP) at different conditions.

	Shear rate (s^−1^)							
Temperature (°C)	1021.38	510.69	340.46	170.23	102.14	51.07	10.21	wt%
**30**	6.05	9.3	12	18.6	24.5	43	165	0.1
**50**	4.5	5.7	7.8	15	23	38	150	
**75**	3.85	5.1	6.75	12	19.5	31	140	
**90**	3.5	4.5	5.85	11.7	16	29	105	
	**1021.38**	**510.69**	**340.46**	**170.23**	**102.14**	**51.07**	**10.21**	
**30**	8.65	13.7	16.4	26.7	35.5	58	245	0.2
**50**	7.05	10.3	13.8	19.5	30	51	225	
**75**	5.15	9.9	11.7	15.6	24	45	210	
**90**	4.5	6.5	9	14.7	22	36	170	
	**1021.38**	**510.69**	**340.46**	**170.23**	**102.14**	**51.07**	**10.21**	
**30**	12.05	20.9	24.8	47.7	58.5	105	440	0.3
**50**	10.65	18.5	21.5	38.7	49.5	87	325	
**75**	9.85	15.4	19.8	34.5	43.5	80	260	
**90**	7.65	13.2	18.6	32.1	41	75	200	
	**1021.38**	**510.69**	**340.46**	**170.23**	**102.14**	**51.07**	**10.21**	
**30**	13	22	31.5	45	67.5	119	450	0.4
**50**	10.95	17.8	23.3	39.9	57.5	92	405	
**75**	9.05	16.3	19.7	34.5	55	80	270	
**90**	8.65	14.5	18.3	32.7	50	70	225	
	**1021.38**	**510.69**	**340.46**	**170.23**	**102.14**	**51.07**	**10.21**	
**30**	14.95	23.2	33.8	53.4	82.5	156	540	0.5
**50**	14.4	21.5	31.4	48.3	76.5	145	495	
**75**	11.65	20.4	26.9	44.1	67.5	127	405	
**90**	10.6	18.3	23.9	40.2	61	111	375	
	**1021.38**	**510.69**	**340.46**	**170.23**	**102.14**	**51.07**	**10.21**	
**30**	31.95	55.3	77	137.7	209.5	389	1645	1.0
**50**	30.05	53.3	74.7	134.1	201	365	1515	
**75**	28.4	48.9	69.5	125.1	189	353	1440	
**90**	26.95	47.4	67.8	122.4	182.5	335	1340

**Table 3:** Guar Gum viscosity (cP) at different conditions.

	Shear rate (s^−1^)							
Temperature (°C)	1021.38	510.69	340.46	170.23	102.14	51.07	10.21	wt%
**30**	4.95	6.7	8.7	14.7	19.5	35	155	0.1
**50**	4.55	5.3	7.35	13.5	17.5	32	145	
**75**	4.25	4.8	6.3	12	16.5	30	135	
**90**	3.95	3.7	5.55	11.1	15	28	100	
	**1021.38**	**510.69**	**340.46**	**170.23**	**102.14**	**51.07**	**10.21**	
**30**	6.85	10.3	13.2	22.2	29.5	48	205	0.2
**50**	5.75	8.8	10.95	19.2	25.5	46	195	
**75**	4.25	6.8	7.95	13.5	24.5	37	165	
**90**	3.45	5.7	7.2	14.4	21.5	36	160	
	**1021.38**	**510.69**	**340.46**	**170.23**	**102.14**	**51.07**	**10.21**	
**30**	10.65	17.7	23.25	35.7	42.5	61	270	0.3
**50**	9.95	15.2	15.75	26.7	36.5	55	240	
**75**	7.7	10.1	12.75	21.6	30.5	49	205	
**90**	6	8.3	7.8	14.7	24.5	44	180	
	**1021.38**	**510.69**	**340.46**	**170.23**	**102.14**	**51.07**	**10.21**	
**30**	16.75	24.7	30.15	43.5	61	101	415	0.4
**50**	13.6	20.5	27.45	34.5	47.5	83	335	
**75**	11.65	18.7	23.1	30.3	44	77	255	
**90**	9.25	15.5	21.15	23.4	33.5	63	195	
	**1021.38**	**510.69**	**340.46**	**170.23**	**102.14**	**51.07**	**10.21**	
**30**	17.85	28.4	36.45	57.3	78	117	410	0.5
**50**	16.6	25.5	30.15	51.6	66.5	101	390	
**75**	14.75	25.2	28.35	43.2	55.5	93	260	
**90**	13.5	23	23.55	39	47	80	245	
	**1021.38**	**510.69**	**340.46**	**170.23**	**102.14**	**51.07**	**10.21**	
**30**	53.35	93.2	126.75	207.3	300	507	1510	1.0
**50**	50.45	92.9	123.3	206.4	289.5	474	1370	
**75**	47.6	80.7	108.45	179.7	250.5	407	1085	
**90**	45.85	73.4	99.45	163.5	227	367	915

**Table 4:** Gum arabic viscosity (cP) at different conditions.

	Shear rate (s^−1^)							
Temperature (°C)	1021.38	510.69	340.46	170.23	102.14	51.07	10.21	wt%
**30**	2.9	5.1	7.35	9.9	15.5	30	150	0.4
**50**	2.65	4.8	5.85	9.6	15	30	145	
**75**	1.75	2.8	4.95	5.1	8	15	70	
**90**	1.3	2	3.75	4.8	7.5	13	65	
	**1021.38**	**510.69**	**340.46**	**170.23**	**102.14**	**51.07**	**10.21**	
**30**	3.6	5.1	7.5	15	24	48	170	0.5
**50**	2.65	5	6	11.7	18.5	37	155	
**75**	2.1	3.9	5.1	7.5	12.5	24	120	
**90**	1.9	3.7	4.5	5.1	8	16	100	
	**1021.38**	**510.69**	**340.46**	**170.23**	**102.14**	**51.07**	**10.21**	
**30**	6.95	12.2	16.2	19.2	33	49	180	1.0
**50**	6.25	11.1	13.5	18.6	27	40	160	
**75**	5.05	6.3	10.95	12.9	20	35	140	
**90**	4.6	4.6	9	10.2	16.5	20	135	
	**1021.38**	**510.69**	**340.46**	**170.23**	**102.14**	**51.07**	**10.21**	
**30**	8.45	13.7	19.05	20.1	35	50	200	5.0
**50**	6.6	12	18	19.5	27.5	42	180	
**75**	6.55	9.5	15	16.2	25	37	170	
**90**	4.5	6.2	12.3	12.9	18	29	140	
	**1021.38**	**510.69**	**340.46**	**170.23**	**102.14**	**51.07**	**10.21**	
**30**	16	19	21	25.2	40	55	220	10.0
**50**	12.5	14	19.05	20.1	32.5	45	200	
**75**	9.5	9.6	18	19.5	27	40	180	
**90**	6.85	8.4	13.2	14.1	20	31	150	
	**1021.38**	**510.69**	**340.46**	**170.23**	**102.14**	**51.07**	**10.21**	
**30**	34.5	37	37.5	40.5	47.5	63	245	15.0
**50**	27	30	28.8	30.9	45	50	220	
**75**	20.5	22	25.5	27.6	30	45	190	
**90**	14.8	16.7	18.75	20.1	26	35	165

**Table 5:** Effect of silica nanoparticle loading on xanthan gum viscosity (cP).

	Shear rate (s^−1^)							
Silica wt%	1021.38	510.69	340.46	170.23	102.14	51.07	10.21	Polymer wt%
**0.1**	5.25	6.5	9.3	16.5	24.5	39	170	0.1
**0.2**	5	6.3	9	15	22.5	36	175	
**0.5**	5	6.2	8.85	14.7	22	36	175	
**0.8**	6	7.2	9.75	18	29	50	190	
**1**	5.25	6.5	9.3	17.1	25	40	185	
	**1021.38**	**510.69**	**340.46**	**170.23**	**102.14**	**51.07**	**10.21**	
**0.1**	16.5	26	36	60	92.5	148	600	0.5
**0.2**	17.75	28	37.5	61.5	95	165	615	
**0.5**	18.5	29.5	39	64.5	97	170	625	
**0.8**	19.75	32.5	42	68.7	100	188	645	
**1**	19.5	32	41.25	67.5	98	180	650	
	**1021.38**	**510.69**	**340.46**	**170.23**	**102.14**	**51.07**	**10.21**	
**0.1**	36	61.5	82.5	142.5	217.5	390	1675	1.0
**0.2**	35	56	75.75	132	205	360	1680	
**0.5**	36.5	63	87	150	225	390	1750	
**0.8**	40	65	88.5	156	235	425	1850	
**1**	42	69	96	163.5	250	460	1880

**Table 6:** Effect of alumina nanoparticle loading on xanthan gum viscosity (cP).

	Shear rate (s^−1^)							
Alumina wt%	1021.38	510.69	340.46	170.23	102.14	51.07	10.21	Polymer wt%
**0.1**	6.25	12.4	18.3	29.1	22.5	42	200	0.1
**0.2**	6.25	12.4	18.3	29.7	22.5	43	200	
**0.5**	5.85	11.2	15.75	28.2	22	41	190	
**0.8**	6.35	12.5	18.45	33.6	23.5	44	205	
**1**	6.75	13	18.75	33.9	25	45	210	
	**1021.38**	**510.69**	**340.46**	**170.23**	**102.14**	**51.07**	**10.21**	
**0.1**	15.5	23.5	30	54	86	145	650	0.5
**0.2**	16.75	24.5	36.75	64.5	97.5	180	750	
**0.5**	16.9	27	37.5	69	100	180	800	
**0.8**	16.5	24	34.5	60	95	170	825	
**1**	16.5	24	33	57	93	155	830	
	**1021.38**	**510.69**	**340.46**	**170.23**	**102.14**	**51.07**	**10.21**	
**0.1**	32	57.5	79.5	139.5	207.5	390	1650	1.0
**0.2**	32.5	58	81	141	225	420	1675	
**0.5**	37.5	62.5	88.5	156	245	470	1800	
**0.8**	35.5	62.5	85.5	150	240	440	1850	
**1**	33	57	81	144	235	390	1860

**Table 7:** Effect of cupric nanoparticle loading on xanthan gum viscosity (cP).

	Shear rate (s^−1^)							
Cupric wt%	1021.38	510.69	340.46	170.23	102.14	51.07	10.21	Polymer wt%
**0.1**	7.5	9.5	13.5	22.5	35	55	205	0.1
**0.2**	6.5	9	12.75	16.5	25	42	200	
**0.5**	6.5	9.1	13.2	21	32.5	60	225	
**0.8**	6	11	16.5	33	55	80	300	
**1**	5.5	9	13.5	27	45	70	325	
	**1021.38**	**510.69**	**340.46**	**170.23**	**102.14**	**51.07**	**10.21**	
**0.1**	18.25	30	42	76.5	105	180	705	0.5
**0.2**	16	27	36	69	92.5	165	700	
**0.5**	18.75	33.5	45	84	115	210	850	
**0.8**	17.5	27.5	40.5	72	100	170	850	
**1**	16	26	34.5	66	90	180	900	
	**1021.38**	**510.69**	**340.46**	**170.23**	**102.14**	**51.07**	**10.21**	
**0.1**	37	64.5	93.75	165	257.5	500	1750	1.0
**0.2**	35	61	82.5	150	225	410	1800	
**0.5**	31.5	57	76.5	141	215	390	1850	
**0.8**	39.5	65	95.25	172.5	265	510	1900	
**1**	32.5	60	81	143.4	218	400	1950

**Table 8:** Effect of silica nanoparticle loading on guar gum viscosity (cP).

	Shear rate (s^−1^)							
Silica wt%	1021.38	510.69	340.46	170.23	102.14	51.07	10.21	Polymer wt%
**0.1**	5.15	7.5	10.05	14.7	22	37	160	0.1
**0.2**	5.15	7.5	10.05	14.7	21	36	160	
**0.5**	5.45	9	11.25	15.9	25.5	41	170	
**0.8**	5.45	9	10.95	15.6	25	39	170	
**1**	5.15	7.5	10.5	15	23	38	175	
	**1021.38**	**510.69**	**340.46**	**170.23**	**102.14**	**51.07**	**10.21**	
**0.1**	22.5	32	45	61.5	97.5	145	550	0.5
**0.2**	23.5	38	48.75	66.9	105	160	595	
**0.5**	23.75	39	50.25	67.5	110	165	600	
**0.8**	22.75	38	48	66	100	150	575	
**1**	24.5	40.5	50.85	69	115	170	700	
	**1021.38**	**510.69**	**340.46**	**170.23**	**102.14**	**51.07**	**10.21**	
**0.1**	71.5	120	168	288	380	725	1750	1.0
**0.2**	73	123.5	168.75	289.5	385	730	1800	
**0.5**	73.25	124	171	291	395	745	1850	
**0.8**	69	117.5	160.5	274.5	379.5	700	1900	
**1**	74.5	127	180	297	400	755	1950

**Table 9:** Effect of alumina nanoparticle loading on guar gum viscosity (cP).

	Shear rate (s^−1^)							
Alumina wt%	1021.38	510.69	340.46	170.23	102.14	51.07	10.21	Polymer wt%
**0.1**	4.75	6.5	8.25	14.7	24	41	170	0.1
**0.2**	5	7.2	9.75	15.3	24.5	43	175	
**0.5**	6	11	15.75	16.5	26	44	180	
**0.8**	5.25	7.8	9.9	15.6	25.5	43	175	
**1**	4.25	6	7.95	14.7	23.5	40	170	
	**1021.38**	**510.69**	**340.46**	**170.23**	**102.14**	**51.07**	**10.21**	
**0.1**	25	41.5	52.5	75	78	161	500	0.5
**0.2**	25.5	42	52.8	76.5	80	167	625	
**0.5**	26	42.2	53.25	78	90	168	635	
**0.8**	26.5	43	54	81	100	170	650	
**1**	25	41	51.75	72	77.5	160	650	
	**1021.38**	**510.69**	**340.46**	**170.23**	**102.14**	**51.07**	**10.21**	
**0.1**	63	108	146.25	270	405	750	1750	1.0
**0.2**	71.25	120	162.75	280.5	412.5	770	1820	
**0.5**	72.5	123.5	169.5	288	427.5	776	1845	
**0.8**	74	126	174	297	440	790	2065	
**1**	73.5	125	172.5	294	435	782	2160

**Table 10:** Effect of cupric nanoparticle loading on guar gum viscosity (cP).

	Shear rate (s^−1^)							
Cupric wt%	1021.38	510.69	340.46	170.23	102.14	51.07	10.21	Polymer wt%
**0.1**	4.5	7	9.75	15.6	23.5	42	160	0.1
**0.2**	5	8	9.9	18.6	24	44	175	
**0.5**	5	8	11.25	19.5	25	46	200	
**0.8**	5	8	10.5	18.9	24.5	45	195	
**1**	4.75	7.5	9.75	16.5	24.5	43	200	
	**1021.38**	**510.69**	**340.46**	**170.23**	**102.14**	**51.07**	**10.21**	
**0.1**	26.25	43.8	54	78	80	160	500	0.5
**0.2**	27.75	46.5	57	87	122.5	180	675	
**0.5**	29.25	46.5	60.75	88.5	123.5	190	700	
**0.8**	27.5	46	56.25	85.5	120	170	720	
**1**	26.5	44	55.5	79.5	119	165	765	
	**1021.38**	**510.69**	**340.46**	**170.23**	**102.14**	**51.07**	**10.21**	
**0.1**	53.5	94	177	300	450	825	1650	1.0
**0.2**	55	95.5	177.75	304.5	452.5	800	1770	
**0.5**	77	137	189	324	480	850	1900	
**0.8**	78	138	189	327	490	855	1950	
**1**	75	133	180	309	465	830	2150

**Table 11:** Effect of silica nanoparticle loading on gum arabic viscosity (cP).

	Shear rate (s^−1^)							
Silica wt%	1021.38	510.69	340.46	170.23	102.14	51.07	10.21	Polymer wt%
**0.1**	6.5	12	15	19.8	32.5	50	185	1.0
**0.2**	6.5	12	15.75	21.6	32.5	52	195	
**0.5**	6.5	10.5	15	21	32.5	50	195	
**0.8**	6.5	12	15.9	22.5	33	52	195	
**1**	6.5	12	15	21	32.5	50	200	
	**1021.38**	**510.69**	**340.46**	**170.23**	**102.14**	**51.07**	**10.21**	
**0.1**	8.5	13.9	19.2	24.6	35.5	51	210	5.0
**0.2**	8.5	13.8	19.05	24	35	50	215	
**0.5**	9	14.2	19.35	25.2	36.5	53	220	
**0.8**	9	14.5	19.5	25.5	37	54	225	
**1**	9	14.1	19.2	24.9	36	52	250	
	**1021.38**	**510.69**	**340.46**	**170.23**	**102.14**	**51.07**	**10.21**	
**0.1**	34.75	38.5	39	42	50	70	265	15.0
**0.2**	42	43.5	52.5	48	60	89	285	
**0.5**	45.5	48	57	57	68	92	305	
**0.8**	36	40	48	46.5	50	85	315	
**1**	47.25	50	58.5	60	70	93	325

**Table 12:** Effect of alumina nanoparticle loading on gum arabic viscosity (cP).

	Shear rate (s^−1^)							
Alumina wt%	1021.38	510.69	340.46	170.23	102.14	51.07	10.21	Polymer wt%
**0.1**	7.25	12.9	16.5	24.3	35.5	55	195	1.0
**0.2**	6.65	12	15.75	22.5	33.5	52	200	
**0.5**	7.25	12.5	16.35	24	35	54	200	
**0.8**	7.25	12	16.2	22.5	34.5	52	210	
**1**	7.25	12	16.2	23.1	34.5	53	215	
	**1021.38**	**510.69**	**340.46**	**170.23**	**102.14**	**51.07**	**10.21**	
**0.1**	9	14.8	19.65	25.8	39	58	210	5.0
**0.2**	8.5	14.3	19.2	24.9	37.5	55	215	
**0.5**	9	14.7	19.5	25.5	38.5	57	225	
**0.8**	8.75	14.5	19.35	25.2	38	56	225	
**1**	9.75	14.9	19.8	26.1	39.5	59	240	
	**1021.38**	**510.69**	**340.46**	**170.23**	**102.14**	**51.07**	**10.21**	
**0.1**	34.5	37.5	39.75	39	45	65	280	15.0
**0.2**	40	42	45	48	57.5	70	300	
**0.5**	45	47.5	48	49.5	67.5	79	305	
**0.8**	39.5	38.5	43.5	42	50	68	315	
**1**	40	40	43.5	45	52.5	69	325

**Table 13:** Effect of cupric nanoparticle loading on gum arabic viscosity (cP).

	Shear rate (s^−1^)							
Cupric wt%	1021.38	510.69	340.46	170.23	102.14	51.07	10.21	Polymer wt%
**0.1**	7.3	13.3	16.5	24.6	37	58	195	1.0
**0.2**	6.7	13	16.65	24	36	57	205	
**0.5**	8.5	13.8	17.25	25.5	38	59	210	
**0.8**	8.5	13.6	17.1	25.2	38.5	60	220	
**1**	8	13.5	16.95	24.9	38	59	225	
	**1021.38**	**510.69**	**340.46**	**170.23**	**102.14**	**51.07**	**10.21**	
**0.1**	10	15	19.8	26.1	39.5	59	225	5.0
**0.2**	10.5	15.1	19.95	26.4	40	60	250	
**0.5**	10.75	15.2	20.25	27	40.5	62	260	
**0.8**	11	15.5	20.4	27.3	41	64	280	
**1**	10.5	15.1	20.1	26.7	40	61	290	
	**1021.38**	**510.69**	**340.46**	**170.23**	**102.14**	**51.07**	**10.21**	
**0.1**	36	39	44.25	48	55	72	275	15.0
**0.2**	42.5	53	54	51	65	90	300	
**0.5**	52.5	79	74.25	84	85	115	340	
**0.8**	50	72.5	67.5	72	80	110	340	
**1**	46	59	60	66	72.5	100	345

**Table 14:** Effect of salinity on the viscosity (cP) of polymers at 10.21 s^−1^.

	Brine weight percentage (wt%)				
	0.05	0.15	0.25	0.35	0.45
Xanthan gum	1550	1550	1450	1425	1400
Guar gum	1450	1400	1350	1325	1300
Gum arabic	250	240	220	210	190

**Table 15:** TGA data for xanthan gum polymer showing temperature and weight progression.

Temp (°C)	Weight (mg)	Temp (°C)	Weight (mg)	Temp (°C)	Weight (mg)	Temp (°C)	Weight (mg)
22.642	7.692	46.943	7.472	64.842	7.158	75.984	6.980
23.009	7.688	47.022	7.471	64.923	7.156	76.069	6.979
24.014	7.678	48.805	7.442	65.008	7.155	77.917	6.952
24.986	7.672	49.952	7.423	65.744	7.142	78.003	6.951
25.042	7.671	50.027	7.422	65.825	7.141	79.937	6.925
26.055	7.665	50.939	7.406	65.903	7.140	80.022	6.924
27.025	7.659	51.015	7.405	65.993	7.138	81.948	6.898
28.013	7.654	51.849	7.390	66.074	7.137	82.036	6.897
28.993	7.648	51.925	7.389	66.156	7.135	83.967	6.873
29.073	7.647	52.003	7.387	66.240	7.134	84.049	6.872
29.928	7.642	52.996	7.370	66.322	7.133	85.991	6.850
30.015	7.642	53.066	7.368	68.798	7.091	86.080	6.849
30.989	7.635	53.989	7.352	68.882	7.090	87.925	6.828
31.082	7.635	54.072	7.350	68.963	7.089	89.865	6.808
32.814	7.623	55.065	7.332	69.045	7.087	90.036	6.806
32.903	7.623	56.071	7.314	69.128	7.086	93.676	6.771
32.993	7.622	56.928	7.299	69.217	7.084	95.783	6.752
33.091	7.621	57.004	7.297	69.298	7.083	95.869	6.751
33.927	7.615	57.948	7.280	69.552	7.079	95.957	6.750
34.019	7.615	58.024	7.279	69.634	7.078	96.042	6.750
34.945	7.608	58.967	7.262	69.717	7.076	96.122	6.749
35.039	7.607	59.050	7.260	69.802	7.075	96.210	6.748
35.963	7.600	59.925	7.245	69.880	7.074	96.294	6.748
36.057	7.599	60.001	7.243	69.967	7.072	96.377	6.747
36.970	7.591	60.964	7.226	70.213	7.068	100.960	6.711
37.060	7.590	61.042	7.224	70.296	7.067	101.210	6.709
37.980	7.582	61.925	7.209	70.377	7.066	108.162	6.665
38.073	7.581	62.002	7.207	72.055	7.039	110.618	6.651
46.000	7.486	62.971	7.190	72.139	7.038	110.701	6.651
46.531	7.478	63.049	7.189	73.809	7.012	113.762	6.636

**Table 16:** TGA data for guar gum polymer showing temperature and weight progression.

Temp (°C)	Weight (mg)	Temp (°C)	Weight (mg)	Temp (°C)	Weight (mg)	Temp (°C)	Weight (mg)
22.666	12.370	55.009	11.871	89.080	11.045	110.922	10.813
23.961	12.350	56.993	11.815	92.920	10.989	111.008	10.813
24.995	12.341	57.069	11.813	93.006	10.988	111.097	10.812
25.043	12.340	58.929	11.760	94.973	10.962	111.186	10.812
26.942	12.325	59.008	11.758	97.982	10.926	111.274	10.811
27.009	12.324	60.968	11.701	98.067	10.925	111.359	10.810
33.019	12.275	61.046	11.699	99.957	10.905	111.439	10.810
34.958	12.257	62.951	11.644	100.042	10.904	111.608	10.809
35.049	12.256	63.033	11.642	104.587	10.861	111.692	10.808
36.973	12.235	64.968	11.587	107.585	10.837	111.780	10.808
37.059	12.234	65.048	11.585	107.670	10.836	111.864	10.807
38.942	12.211	66.922	11.533	108.959	10.827	111.952	10.807
39.025	12.209	67.001	11.530	109.041	10.826	112.038	10.806
40.944	12.182	68.979	11.477	109.128	10.825	112.123	10.806
41.031	12.181	69.063	11.474	109.216	10.825	112.207	10.805
42.946	12.150	70.968	11.424	109.298	10.824	112.291	10.805
43.034	12.149	71.050	11.422	109.385	10.824	112.373	10.804
44.964	12.113	72.970	11.373	109.469	10.823	112.466	10.803
45.039	12.112	73.061	11.370	109.554	10.822	112.548	10.803
46.930	12.073	76.835	11.280	109.644	10.822	112.635	10.802
47.004	12.072	76.919	11.278	109.730	10.821	112.720	10.802
48.989	12.027	77.000	11.276	109.818	10.820	112.805	10.801
49.066	12.025	79.885	11.213	109.902	10.820	112.886	10.801
50.946	11.979	79.967	11.211	109.984	10.819	112.977	10.800
51.013	11.977	80.052	11.209	110.071	10.819	113.061	10.800
52.974	11.926	84.981	11.113	110.159	10.818	113.147	10.799
53.047	11.924	85.066	11.112	110.242	10.818	113.232	10.799
54.853	11.875	86.938	11.079	110.328	10.817	113.324	10.798
54.933	11.873	87.027	11.078	110.752	10.814	113.406	10.798
54.991	11.871	88.989	11.046	110.839	10.814	113.491	10.797

**Table 17:** TGA data for gum arabic polymer showing temperature and weight progression.

Temp (°C)	Weight (mg)	Temp (°C)	Weight (mg)	Temp (°C)	Weight (mg)	Temp (°C)	Weight (mg)
21.853	11.528	56.402	11.015	92.778	10.441	110.661	10.254
22.940	11.507	56.483	11.013	92.868	10.440	110.739	10.253
24.089	11.497	58.415	10.973	94.812	10.417	110.826	10.252
24.154	11.497	58.494	10.972	97.777	10.384	110.913	10.252
26.238	11.481	60.538	10.931	97.862	10.383	110.994	10.251
26.322	11.481	60.625	10.930	99.724	10.363	111.080	10.250
32.541	11.429	62.603	10.891	99.810	10.362	112.011	10.241
34.452	11.409	62.687	10.890	104.310	10.315	112.096	10.241
34.538	11.408	64.686	10.852	107.275	10.286	112.179	10.240
36.409	11.387	64.770	10.851	107.354	10.285	112.267	10.239
36.497	11.386	66.701	10.816	108.624	10.273	112.351	10.238
38.311	11.362	66.781	10.815	108.709	10.272	112.442	10.238
38.393	11.361	68.810	10.780	108.796	10.272	112.523	10.237
40.233	11.334	68.889	10.778	108.884	10.271	112.611	10.236
40.315	11.332	70.828	10.745	108.964	10.270	112.689	10.236
42.166	11.302	70.913	10.744	109.052	10.269	112.776	10.235
42.252	11.301	72.857	10.713	109.134	10.268	112.860	10.234
44.137	11.267	72.945	10.711	109.222	10.267	112.945	10.233
44.217	11.265	76.765	10.653	109.303	10.267	113.026	10.232
46.070	11.230	76.845	10.651	109.387	10.266	113.112	10.232
46.144	11.229	76.934	10.650	109.472	10.265	113.197	10.231
48.140	11.188	79.818	10.608	109.559	10.264	113.285	10.230
48.212	11.187	79.898	10.607	109.643	10.263	113.365	10.229
50.132	11.147	79.988	10.606	109.731	10.263	113.448	10.229
50.212	11.145	84.908	10.539	109.813	10.262	113.534	10.228
52.221	11.103	84.987	10.538	109.898	10.261	113.626	10.227
52.298	11.101	86.852	10.514	109.980	10.260	113.703	10.226
54.174	11.061	86.936	10.512	110.404	10.256	113.788	10.226
54.251	11.060	88.885	10.488	110.489	10.255	113.823	10.226
54.334	11.058	88.971	10.487	110.570	10.255	113.959	10.224

## 2 Experimental Design, Materials and Methods

### 2.1 Materials

Xanthan Gum and Guar Gum were purchased from Ojota Chemical Market in Lagos while Gum Arabic was purchased from Panteka Market in Kaduna State. Silica and alumina nanoparticles were purchased from Sigma Aldrich while cupric nanoparticle was purchased from BDH AnalaR. Sodium chloride was used to prepare the brine solution.

### 2.2 Characterization of polymers and nanoparticle

High-resolution micrographs were obtained for the nanoparticles using Quanta SEM 450 Equipment. Scanning was done at a spot size of 3–5 and a voltage range of 3–5 kV. FTIR analysis was done for the three polymers using Brucker Vertex 80v Instrument with an Attenuated Total Reflectance (Type A225/Q) in transmittance mode, and the analysis was done with the OPUS 7.0 software. The sample time scan and background time scans were both 64 scans. The spectra were taken between 400 and 4000 cm^−1^. TGA was done using TA Instrument Q6000 (SDT V20.9 Build 20) to examine the thermal stability of the polymers. Thermal stability was measured based on weight change using a horizontal dual beam with automatic beam growth compensation. The gas flux and heating range used for the measurements were 100 mL/min and 10 °C/min respectively, and between a temperature range of 20–120 °C.

### 2.3 Preparation of polymer and nanocomposite fluids

Polymer fluids were prepared by adding measured quantities of the required polymer directly to deionized water according to standard method API 63 – “Practices for evaluation of polymers used in EOR.” Gum Arabic fluid was prepared to the following weight percentages: 0.4, 0.5, 1, 5, 10 and 15% w/w. Xanthan Gum and guar gum were prepared to the following weight percentages: 0.1, 0.2, 0.3, 0.4, 0.5 and 1% w/w. The viscosity of Gum Arabic varies widely from the other two polymers; hence, lower weight percentages could not be prepared for gum arabic. The preparation of polymer nanocomposites was also prepared using the direct addition method. Xanthan Gum and Guar Gum were allowed for 24 h before measurements were taken while Gum Arabic was allowed for 48 h. This was to ensure complete dissolution.

### 2.4 Measurement of rheological properties of polymers at different temperatures

A Model 800 OFITE viscometer was used to obtain dial readings at different shear rates which were then converted to viscosity values. Desired temperatures (30, 50, 75 and 90 °C) of polymer solutions were achieved using the heating mantle of the viscometer. The viscosity values were calculated from the dial readings using the formula in Eq. (1).

(1) $$\eta =KF\frac{\theta }{\text{RPM}}$$

where η is the viscosity in centipoise, θ is the dial reading obtained from the viscometer, *K* is the machine constant of the Rotor – Bob combination (R1B1) and *F* is the spring factor. *K* value for the R1B1 is 300 while its *F* value is 1.

## Declaration of Competing Interest

None.
